# Effects of Municipal Solid Waste on Planting Properties and Scouring Resistance of Vegetation Concrete (Wuhan, China)

**DOI:** 10.3390/ijerph19138143

**Published:** 2022-07-02

**Authors:** Liulin Kong, Xiaomei Wang, Wencheng Guo, Yongcheng Zhang

**Affiliations:** 1 Faculty of Engineering, China University of Geosciences, Wuhan 430070, China; kongliulin@cug.edu.cn; 2 China Academy of Building Research Institute, Beijing 100029, China; guowencheng@cabrtech.com; 3 Beijing Glory PKPM Technology Co., Ltd., Beijing 100013, China; 4 Department of Engineering Management, Huaiyin Institute of Technology, Huaian 223003, China; cquzhych@hyit.edu.cn

**Keywords:** municipal solid waste, vegetation concrete, seed germination, plant height, scouring resistance

## Abstract

Vegetation concrete (VC) laid as a reinforcement base and covered by a soil layer with vegetation has been increasingly used to beautify the landscape, reduce environmental pollution and control stormwater runoff. In this study, the effects of municipal solid waste (MSW) on vegetation characteristics of modified VC were tested under different mix compositions. We first explored the effects of the mixed concrete environment on *Festuca elata* and perennial *Ryegrass* for 60 days. Then, the influence of various MSW contents added to different percentages of cement on scouring resistance of VC was examined. The experimental results revealed that the germination rates and plant heights of both species decreased with the increase in concrete content. Considering the scouring resistances, the optimal mix proportion of MSW-modified VC was recommended as No. 25, with 5% KW fertilizer, 8% cement and 0.5% wheat straw in this study. Furthermore, adding a small amount of fallen leaves or silica fume to VC can promote the growth of both species to some extent, although these additions had an inverse effect on the scouring resistances. The results contribute to beneficial knowledge for future research on the feasibility of the use these species with VC technology for slope ecological restoration.

## 1. Introduction

In the field of slope disaster management and ecological protection, a number of measures have been taken, and new techniques have been developed [1]. One of the fastest and most environmentally friendly techniques to avoid and remedy runoff on a slope is to vegetate it [2]. Research has shown that the natural soil lacks scouring resistance and therefore cannot be directly used as overlaying soil for plants [3]. Researchers (e.g., [4], Cao, Wang [5,6]) have used cement (Portland cement (PC) and sulfoaluminate cement (SAP)) as solidified agents to improve the scouring resistance of plants because its hydration and hardening can produce a mechanically stable form. For integration of vegetation with concrete, porous concrete is used as the medium so that water, air, soil and roots can pass freely and vegetation can germinate and root itself through the porous concrete frame and into the underlying soil stratum [7]. Vegetation concrete (VC) laid as a reinforcement base and covered by a soil layer with vegetation has been increasingly used to beautify the landscape, reduce environmental pollution and control stormwater runoff [7,8]. It is crucial to understand the effects of different concrete contents on seed germination and seedling establishment so that the appropriate concrete content range in for application of VC can be determined [9]. Chen et al. [9] evaluated the impacts of concrete content on seed germination and seedling establishment and found that the optimum ratio of concrete content as a stabilizing agent was 8%. However, plant roots require adequate moisture and nutrients to grow and extend, which cannot be satisfied by VC. In addition, due to the weakly alkaline environment of VC, plants cannot grow properly on it, as the most suitable soil for plant growth is slightly acidic. Thus, many researchers began to use low-basicity cement or to add admixtures such as fly ash and slag to reduce the alkalinity of vegetation concrete. Yan et al. [10] examined the influence of anhydrite on mechanical the properties and alkalinity of sulfoaluminate cement clinker. Martin et al. [11] investigated the properties of calcium sulfoaluminate (CSA) cements blended with limestone. Other researchers have added fertilizers or soil amendment into VC to provide nutrition for plant growth. For instance, Yuan et al. [12] studied a new and improved multi-aggregate ecological concrete (IMAEC) revetment treatment by adding activated carbon and other materials to the VC, which resulted in higher water purification performance than traditional ecological concrete (TEC) revetment treatment. Gong et al. [13] investigated the effects of carbamide as a fertilizer added to planting concrete on properties of cement and evaluated the adaptability of sulfoaluminate cement (SAC) concrete for plant growth. 

However, the development of VC composite is still in its infancy, and there is little available published literature for direct review [7]. Therefore, there is a practical necessity to propose a novel, cost-effective and eco-friendly VC modification approach that retains adequate scouring resistance. Owing to growing environmental concerns, the establishment of sustainable materials and practices is important for future societies [14,15]. Municipal solid waste (MSW), i.e., kitchen waste (KW), wheat straw, fly ash, silica fume and fallen leaves, provides an alternative for VC modification. Current research shows that the organic content (such as kitchen and garden waste) of MSW can be used as a soil amending agent to help increase plant growth and improve plant health [16]. A total of 1300 million tons/year (Mt/year) of MSW is generated at a rate of 1.2 kg/capita/day [17], and this considerable quantity of MSW is anticipated to rise to 2200 Mt/year by 2025 [18], representing the most abundant type of waste in China [19]. Harmless MSW disposal methods need to be developed to satisfy the economic development situation [20]. Thus, the addition of MSW to VC is not only prominent in improving soil properties as a soil conditioner but also economical and practical in terms of environmental impact and cost.

MSW is the largest structural element of a municipal landfill [21] and has direct influence on the stability of sanitary landfills and the integrity of lining systems [22]. Thus, considerable attention has been paid to the engineering properties of MSW. Related studies have mainly focused on the effects of factors such as waste type, composition, decomposition, moisture conditions, test methods, unit weight, and particle size and shape on the comparative or shear strength behaviors of MSW [21,23,24,25,26,27,28]. For example, Liu et al. [29] combined fly ash, ferrous sulfate and vermiculite vinyl acetate emulsion to enhance the physical and chemical characteristics of vegetation substrate. Cheng et al. [30] added fly ash and silica fume to VC as admixture and supplementary cementing materials, and results showed that the effects of silica fume on strength, porosity and pH value were greater than those of fly ash. Hu et al. [31] combined palm fiber, silicon fume and entraining agent as admixture to increase the frost resistance of VC with a mass loss rate of 2.81% and relative elastic modulus of 84.27%. Limited studies have been reported on the vegetation performance of MSW (such as fly ash, silica fume, etc.)-modified VC. For instance, Zhao et al. [8] examined the effects of fly ash slag and superplasticizer on VC, and experimental results showed that *Chloris truncata* was better adapted to the VC environment than *Elymus scaber* and *Themeda trianda*, although *Elymus scaber* thrived with a remarkable coverage with high fly ash content and *Themeda trianda* preferred a lower fly ash content. Bao et al. [32] used self-made reducing cement and alkaline admixture with silica fume as the main component; the effect of this admixture on vegetation growth was investigated in a small-scale field for ten weeks. Kim and Park [33] investigated the void ratio, compressive strength, freeze–thaw resistance, plant growth and water purification properties of porous VC formed using the industrial byproducts blast furnace slag powder and blast furnace slag aggregates. However, most work has mainly focused on the effect of one parameter, such as fly ash or silica fume, on the performance of VC, although it is affected by a large number of parameters [34]. Moreover, the combined effects of multiple parameters on the performance of VC remain less known.

The aim of the study presented in this paper was to propose a cost-effective and eco-friendly VC modification approach, retaining adequate scouring resistance. Therefore, extensive experiments were conducted on the addition of MSW to the ecological base material of VC. Comparative experiments were designed and tested under different mix compositions by controlling other environmental factors and varying MSW contents with different treatments. Then, based on the experimental results, the effects of the mixed concrete environment on the selected grass species were explored. We first investigated the growth characteristics (i.e., average seed germination and grass height) of two plants (*Festuca elata* and perennial *Ryegrass*) on VC; a total of 30 mix designs containing different percentages of kitchen waste, wheat straw, fly ash, silica fume and fallen leaves, along with different amounts of Portland cement, were prepared and used to produce VC specimens. We them performed a detailed factor analysis of effects of the addition contents on seed germination and growth properties. Furthermore, the influence of various addition contents with different percentages of cement (4%, 8% and 12%) on scouring resistance of VC was examined. Finally, conclusions were drawn.

## 2. Materials and Methods

### 2.1. Raw Materials and Mixture Proportions

#### 2.1.1. Soil

**Figure 1:**
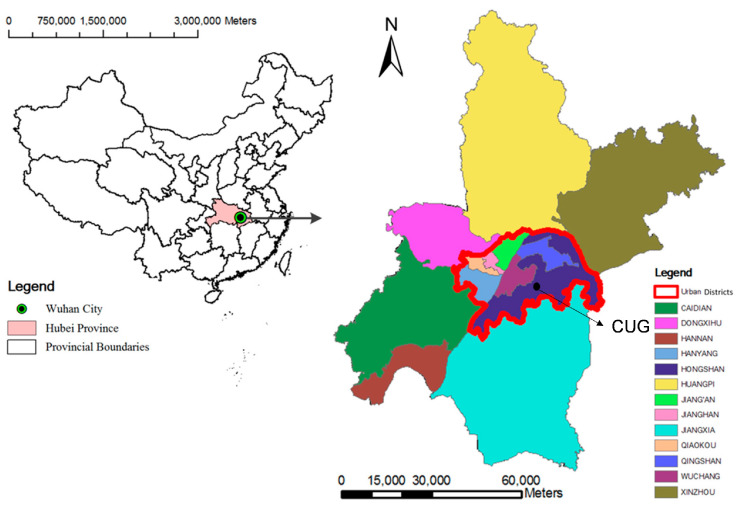
Location and administrative division of the study area.

The soil used for this study was collected from Nanwang mountain slope (below the surface layer to about 20 cm) at China University of Geosciences (CUG, Wuhan, China) (Figure 1) and classified as clay according to the Unified Soil Classification System (USCS). The soil was screened to an aggregate gradation of 2.5 mm. The physical performance indices of the soil after drying are listed in Table 1. As shown in Table 1, the relative density of the soil was 2.5, and its plastic limit and liquid limit were 17.6% and 32.3%, respectively. According to the standard Proctor compaction test, the soil had a maximum dry density (MDD) of 1.73 g/cm^3^, which corresponds to the optimum moisture content (OMC) of 15.6%.

#### 2.1.2. Compound Portland Cement

The compound Portland cement used in this study was produced in Tangshan, China, and had a strength of 32.5 Mpa after an initial setting time of 2 h and final setting time of 6.5 h. According to the experimental results reported in [9], the optimum ratio of concrete content for seed germination and seedling establishment was 8%. Thus, three different percentages of cement were selected: 4%, 8% and 12%.

#### 2.1.3. Admixtures

(1) Organic fertilizer made of kitchen waste (KW)

The household KW fertilizer used in this research consisted of vegetables, peel, fish viscera, rice, lard, etc., with high water content, a low C/N ratio and low porosity. As research showed that KW fertilizer can be used to increase acidic soil pH and increase soil carbon content [20], and the suitable soils for the growth of the two selected grass species are slightly acidic, we considered the ratio of KW to be less than 15%.

(2) Silica fume

The silica fume used in this study was produced in Henan, China. According to [35], the optimum addition percentage of silica fume to significantly improve the compressive strength of concrete is 5%. Silica fume with weight percentages of 4%, 6%, and 8% were employed in the concrete mix design.

(3) Fly ash

The fly ash used in this study was produced by Shandong Hengyuan New Material Co., Ltd. and usually used as a supplementary cementing material to reduce the amount of cement, and weight percentages of 10%, 20% and 30% were employed in the concrete mix design.

(4) Wheat straw

The waste of wheat straw contains high amounts of silica, especially in the leaves [36]. The weight percentages of wheat straw were designated as less than 1.5% in the concrete mix design. The wheat straw used in this study was produced in Henan, China. Before sample preparation, the epidermis of the wheat straw was stripped, and the stem nodes were cut off with the required reinforcement length of 2 cm.

(5) Fallen leaves

According to [37], more than 30 million tons of green waste are produced every year in China, with fallen leaves and tree pruning waste representing dominant forms. Another study showed that bulked aerobic composting with fallen leaves has potential for kitchen waste disposal [19], then fallen leaves and KW fertilizer may have combined effects on the vegetation performance of VC. Therefore, we selected fallen leaves as soil amendments with the same comparable content ratio as that of wheat straw (≤1.5%).

#### 2.1.4. Plants Seeds

Two common plants (i.e., *Festuca elata* and perennial *Ryegrass*) for slope protection were used to measure the adaptability between overlaying soil and plants; the pH of suitable soil for *Festuca elata* is 4.7~8.5, and that for *Ryegrass* is 6~7. These two plant seeds were purchased from Taobao and produced in Jiangsu, China.

### 2.2. Specimen Preparation and Test Procedure

#### 2.2.1. Mix Proportion

In this research, five types of MSW used as ecological slope-protected base material for VC (i.e., kitchen waste, wheat straw, fly ash, silica fume and fallen leaves) were added to three different percentages of Portland cement (4%, 8% and 12%) under different mix compositions. A total of 30 mixtures of VC specimens were designed, which are listed in Table 2.

#### 2.2.2. Planting Tests

**Figure 2:**
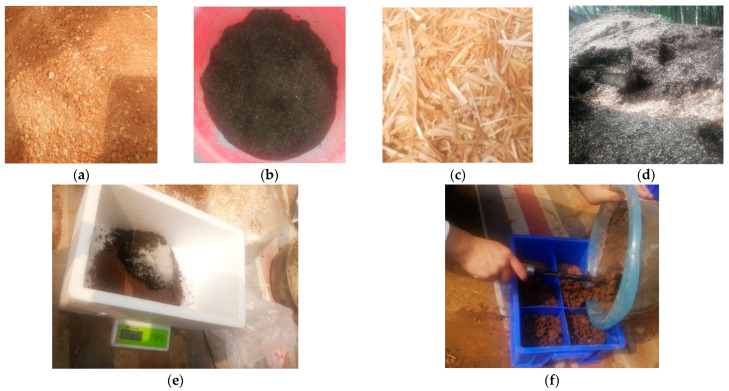
MSW used for VC sample production. (**a**) Soil; (**b**) KW fertilizer; (**c**) wheat straw; (**d**) fallen leaves; (**e**) mixing; (**f**) filling the mold.

**Figure 3:**
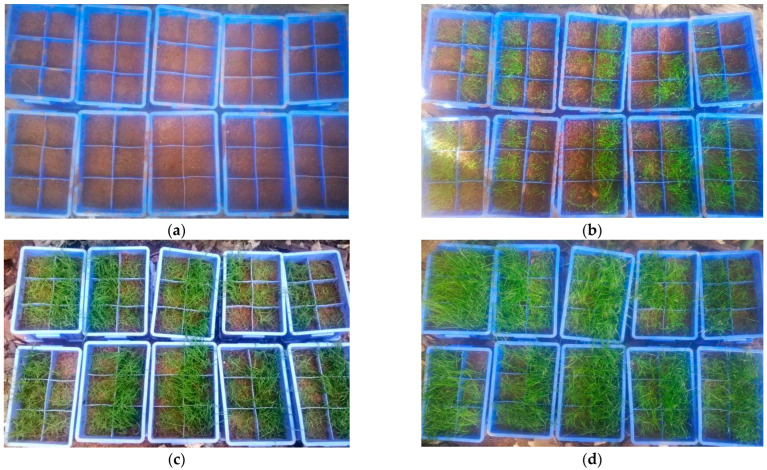
Actual effect pictures of adaptability investigation. (**a**) Ecological concrete covering soil with seeds; (**b**) sowing seeds for 10 days; (**c**) sowing seeds for 30 days; (**d**) sowing seeds for 60 days.

The procedure for fabricating MSW-modified VC is presented in Figure 2. The substrates of VC were first prepared according to the proportions, and the samples were fabricated by filling the substrates into the incubators with a thickness of 8 cm. Then, 150 seeds (N_0_) of each grass were sown evenly into the upper layers of the samples, which were covered with an approximately 1 cm thick layer of substrate to prevent drying. Furthermore, the samples were labelled and placed in a curing room. Finally, an appropriate amount of water was sprayed once every 3 days for 60 days. The growth characteristics of the vegetation concrete grass species were monitored for 60 days, during which the average seed germination and grass height were observed at the specified planting age (e.g., 5 d, 10 d, 15 d, 20 d, 30 d, 40 d, 50 d and 60 d) (as presented in Figure 3). Then, seed germination rates were calculated.

#### 2.2.3. Scouring Tests

**Figure 4:**
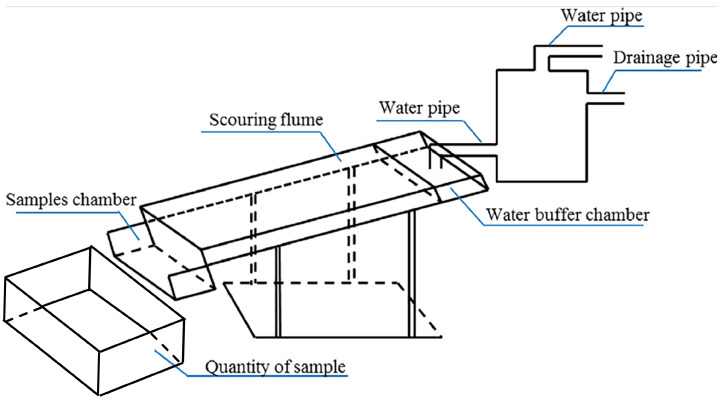
Setup for SRS test.

**Figure 5:**
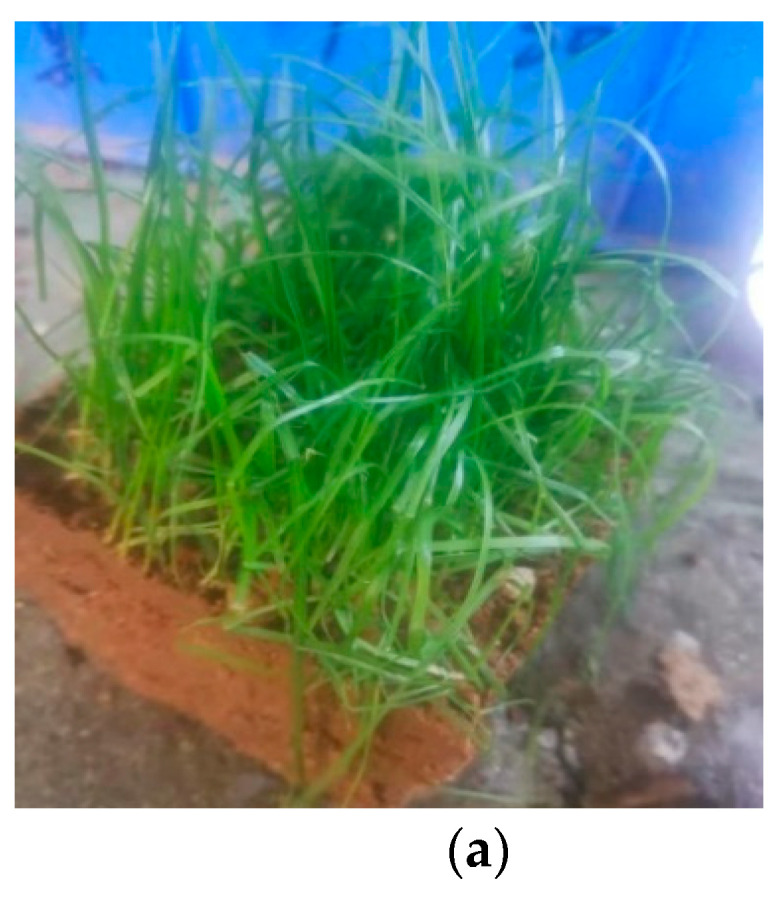

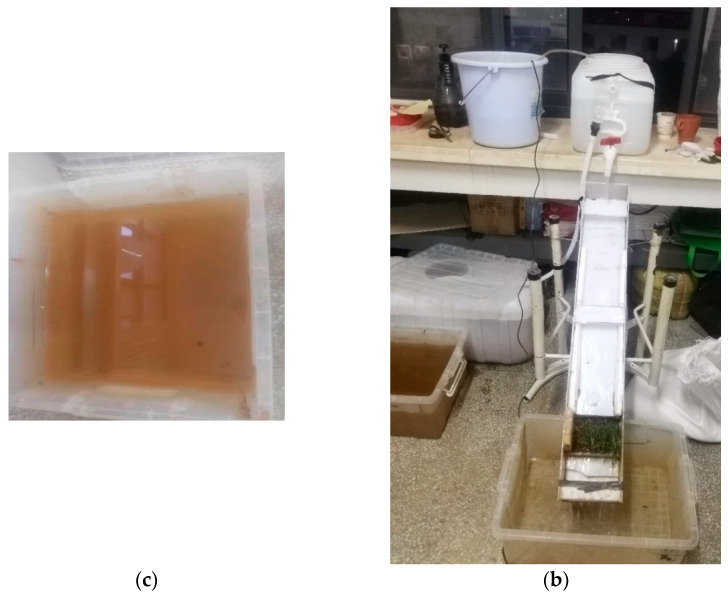
SRS test configuration: (**a**) VC sample with plants; (**b**) test process; (**c**) washed outflows.

The scouring resistance of soil (SRS) was measured in accordance with [38], and the test set-up is presented in Figure 4. When the planting age of 60 days was reached, VC samples with plants were demolded from the incubators, and the integrity of the root system was maintained without disturbance to the greatest extent possible (as shown in Figure 5a). Then, the VC samples were placed in the ‘samples chamber’ and exposed to a flow rate of 2 L/min on a slope of 30° over a period of 30 min in the ‘scouring flume’ (as shown in Figure 5b). A box for ‘quantity of sample’ placed at the foot of the slope was used to collect all the washed outflows. After 30 min, the outflows were allowed to stand for 2 h (as shown in Figure 5c) so the sediments could be thoroughly precipitated. Finally, the box was placed in a drying oven at 105 ± 2 °C to obtain the actual dry weight of the sediments.

## 3. Test Results and Discussion

Research has shown that seed germinations and plant growth are critical to community development, as well as the structure and sustainability of slopes [39,40]. Therefore, appropriate conditions should be developed for seed germination and plant growth to protect a slope and restore its ecological function, both of which are influenced by multiple environmental factors in the field.

### 3.1. Effect of MSW on Planting Properties

#### 3.1.1. Basic Properties

The seed germinations of the two grasses were recorded as N_1_ and N_2_, respectively (Table 3). To determine grass length, five representative grasses for each mixture were measured with a ruler in each part of the test area, and their length was averaged (as listed in Table 4 and Table 5).

The equations employed to obtain the germination ratio (GR) of the two grasses were as follows:

GR_1_ = N_1_/N_0_ × 100% (1)

GR_2_ = N_2_/N_0_ × 100% (2)

where N_0_ is the total amount of grass seeds.

As shown in Table 3, most mixes had high germination rates in the first 15 days and low germination rates in the last 15 days. Moreover, it took 7.3 days and 11.1 days to reach a 50% germination rate for *Ryegrass* and *Festuca elata*, respectively. At the end of the 30-day observation period, the average germination rate of *Ryegrass* and *Festuca elata* reached 91.7% and 80.9%, respectively, which may indicate that the designed mixtures were suitable environments for plant growth.

As shown in Table 4 and Table 5, higher grass heights for all seeds were observed in the first 30 days. At the end of the 30-day observation period, the average grass heights of *Ryegrass* and *Festuca elata* reached 12.7 cm and 10.4 cm, respectively, reaching, 13.9 cm and 12.8 cm, respectively, after 60 days, which may indicate that the designed mixtures were suitable environments for plant growth.

#### 3.1.2. Effect of Cement Content on Seed Germination and Plant Height

**Figure 6:**
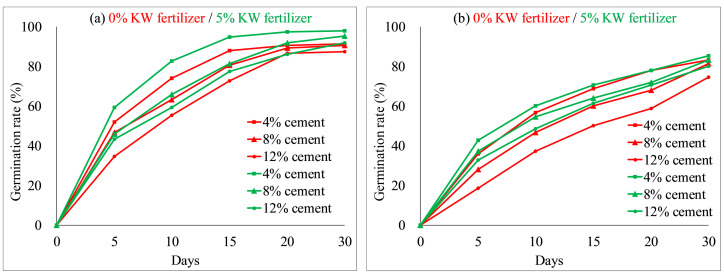
Seed germination rates related to different percentages of cement: (**a**) *Ryegrass*; (**b**) *Festuca elata*.

**Figure 7:**
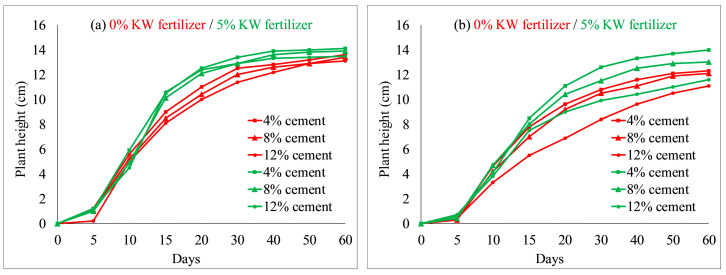
Plant heights related to different percentages of cement: (**a**) *Ryegrass*; (**b**) *Festuca elata*.

The effects of concrete content on seed germination and plant height of each species were tested by comparative experiments, as illustrated in Figure 6 and Figure 7. Other environmental factors (soil and KW fertilizer) were controlled, with the concrete content varied in three treatments (4%, 8% and 12%); red lines denote a comparative group, and green lines represent another group. As shown in Figure 6 and Figure 7, the germination rates and plant heights of each species decreased when concrete content increased from 4 to 12% in the comparative environments. For instance, on day 30, with 5% KW fertilizer, *Ryegrass* achieved a germination rate of 98% and that of 85% for *Festuca elata* both at 4% concrete content; while at 12% concrete content, germinations of *Ryegrass* and *Festuca elata* decreased to 92% and 80%, respectively. Similar results have been reported in the literature. For example, Chen et al. [9] reported that seed germination of *F. arundinacea* and *M. sativa* decreased with increased concrete content. In addition, on day 30 with 5% KW fertilizer, the plant height of *Ryegrass* and *Festuca elata* reached 14.1 cm and 14.0 cm with 4% concrete, respectively; and those decreased to 13.5 cm and 11.6 cm at 12% concrete content, respectively. The test results showed the concrete content had greater effects on *Festuca elata*. Comparing the red and green lines with the same labels, germination rates and plant heights of each species increased when KW fertilizer content increased from 0 to 5% with the same cement content.

#### 3.1.3. Effect of MSW on Grass Heights

**Figure 8:**
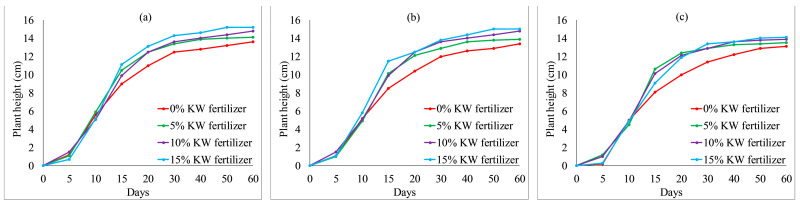
*Ryegrass* height relative to KW fertilizer content with different percentages of cement: (**a**) 4% cement; (**b**) 8% cement; (**c**) 12% cement.

**Figure 9:**
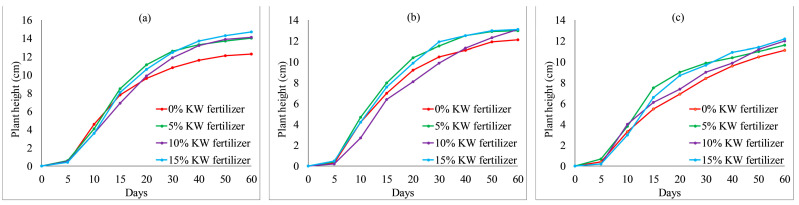
*Festuca elata* height relative to KW fertilizer content with different percentages of cement: (**a**) 4% cement; (**b**) 8% cement; (**c**) 12% cement.

The growth properties of *Ryegrass* and *Festuca elata* affected by KW fertilizer are shown in Figure 8 and Figure 9, respectively. In general, after 15 days, with an increase in KW fertilizer content from 0 to 15%, the plant heights of *Ryegrass* were increased. When KW fertilizer content increased from 5 to 15%, the plant heights of *Festuca elata* fluctuated from day 0 to day 60. The overall difference was not obvious, although all heights were higher than with 0% KW fertilizer content after 40 days, as shown in Figure 9. We conclude that KW fertilizer can be used for VC. Moreover, *Ryegrass* preferred a high KW fertilizer content for a remarkable plant height, whereas *Festuca elata* thrived with any KW fertilizer content.

**Figure 10:**
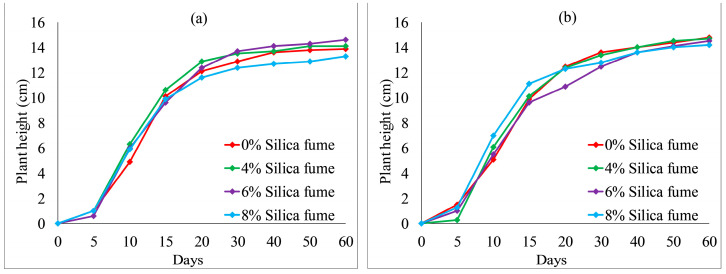
*Ryegrass* height related to silica fume combined with: (**a**) 5% KW fertilizer content; (**b**) 10% KW fertilizer content.

**Figure 11:**
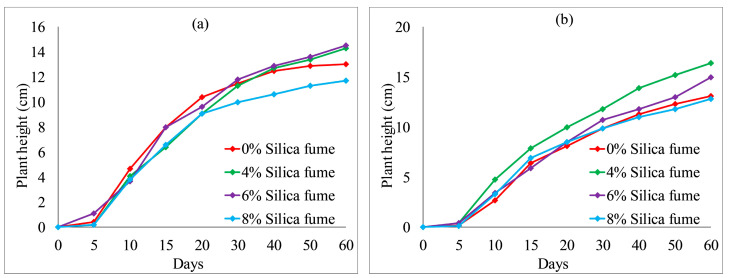
*Festuca elata* height relative to silica fume content combined with: (**a**) 5% KW fertilizer content; (**b**) 10% KW fertilizer content.

Considering the trade-off between mechanical property and vegetation characteristics of VC, the percentage of the cement content in the following VC samples was controlled as 8%. Figure 10 and Figure 11 show the effect of silica fume content on the plant height. With the increase in silica fume content from 0 to 8%, the plant heights of each group fluctuated from day 0 to day 25. The plant heights were the lowest when the silica fume content reached the highest concentration (8%) in each group at 60 days. However, when the ratio of KW fertilizer content was 5%, with an increase in silica fume content from 0 to 6%, the plant heights of both species were increased from day 30 to day 60, as presented in Figure 10a and Figure 11a. When the ratio of KW fertilizer content increased to 10%, with an increase in silica fume content from 4 to 8%, the plant heights of *Festuca elata* were decreased obviously after 20 days, as shown in Figure 11b. This may indicate a combined action of KW fertilizer and silica fume content on plant height, so the addition of silica fume content should be considered together with the KW fertilizer content.

**Figure 12:**
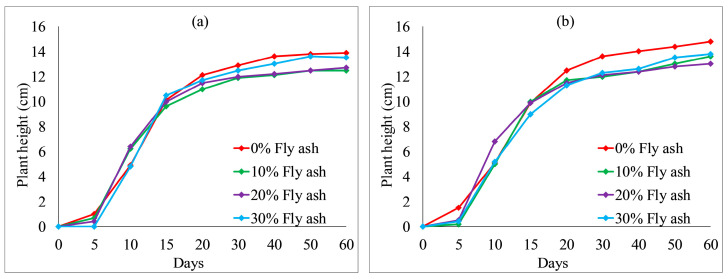
*Ryegrass* height relative to fly ash content combined with: (**a**) 5% KW fertilizer content; (**b**) 10% KW fertilizer content.

**Figure 13:**
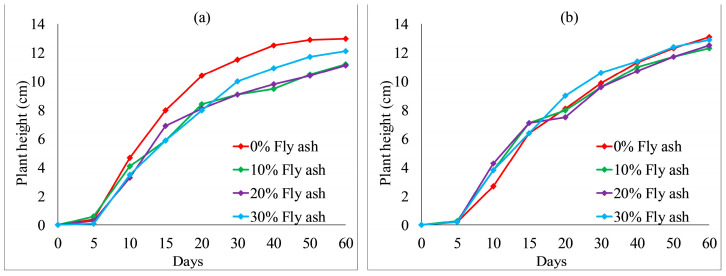
*Festuca elata* height relative to fly ash content combined with: (**a**) 5% KW fertilizer content; (**b**) 10% KW fertilizer content.

**Figure 14:**
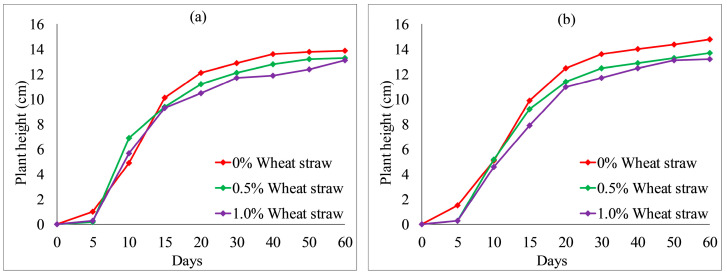
*Ryegrass* height relative to wheat straw content combined with: (**a**) 5% KW fertilizer content; (**b**) 10% KW fertilizer content.

**Figure 15:**
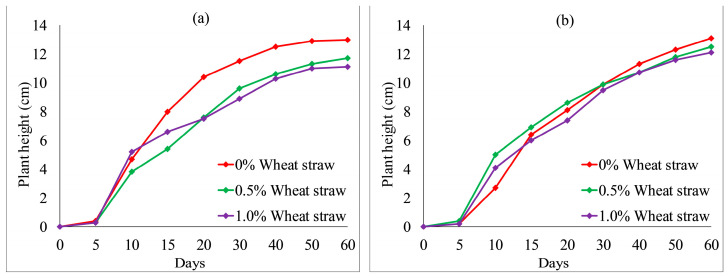
*Festuca elata* height relative to wheat straw content combined with: (**a**) 5% KW fertilizer content; (**b**) 10% KW fertilizer content.

**Figure 16:**
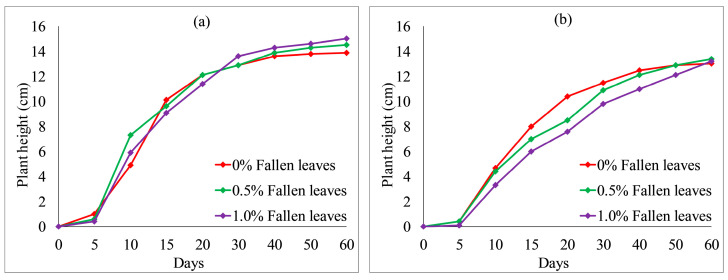
Plant heights relative to fallen leaves content combined with 5% KW fertilizer: (**a**) *Ryegrass*; (**b**) *Festuca elata*.

Figure 12 and Figure 13 show the effect of fly ash content on plant height. With an increase in fly ash content from 10 to 30%, the plant heights of each group also fluctuated. However, the overall difference was not obvious. After 20 days, the plant heights were the highest when no fly ash content added to each group, as shown in Figure 12 and Figure 13a. The test results provide evidence of an inhibition between fly ash content and plant height. Figure 14 and Figure 15 illustrate the effect of wheat straw content on plant height. With an increase in wheat straw content from 0 to 1.0%, the plant heights of *Ryegrass* decreased after 15 days, as illustrated in Figure 14. As shown in Figure 15a, the plant heights of *Festuca elata* decreased after 15 days when the ratio of KW fertilizer content was 5%. The ratio of KW fertilizer content was increased by 10%, and there was no obvious inhibitory effect before day 30 when the wheat straw content was increased from 0 to 1.0%, as presented in Figure 15b. On the whole, after 30 days, the test results show an inverse relation between wheat straw content and plant height. The plant heights were the highest when there was no wheat straw content. This may indicate that the plant heights mainly rely on the properties of the soil added to concrete and KW fertilizer, and the additions of fly ash and wheat straw content have an inhibitory effect on plant heights. As illustrated in Figure 16a, with an increase in fallen leaves content from 0 to 1%, the plant heights of *Ryegrass* increased from day 30 to day 60. As shown in Figure 16b, there was an obvious inhibitory effect on the plant heights of *Festuca elata* when the fallen leaves content increased to 1.0% from day 10 to day 45. However, there was no significant difference in the plant heights on day 60. In general, adding a small amount of fallen leaves to VC can promote the growth of *Ryegrass* to some extent.

In general, MSW contents, such as KW fertilizer, fly ash and wheat straw, have an obvious effect on the germination rates and plant heights of both species. In our study, the former promoted them, whereas the others restrained them. Fallen leaves content slightly promoted germination rates and plant heights, whereas silica fume content had increased them to some extent. The combined action of silica fume content and KW fertilizer content had a positive effect on the germination rates and plant heights of both species with a moderate addition of silica fume (<4%). This indicates that the combined admixtures lowered the pH of VC to some extent, improving the plant development environment. The combined action of fly ash content and KW fertilizer content had an inhibitory effect on the germination rates and plant heights of both species, as did the combined action of wheat straw content and KW fertilizer content. Researchers have reported that the addition of fly ash content [7,36] or KW fertilizer (a raw material for biochar [8]) [20] decreased the pH of VC, which promoted vegetation growth. Our test results indicate that the combination of fly ash content and KW fertilizer content decreased the pH of VC to a certain extent, which was beyond the suitable pH of the soil for vegetation growth of *Ryegrass* and *Festuca elata*. 

### 3.2. Scouring Resistance of Soil (SRS)

SRS can be determined according to the following Equation (3) [3]:

SRS = f × t/W(3)

where f is the water flow rate (L/min), t is the scouring time (min) and W is the weight of the oven-dried sediment (g). Higher SRS values of samples represent a higher resistance to scouring. The SRS test results are listed in Table 6.

**Figure 17:**
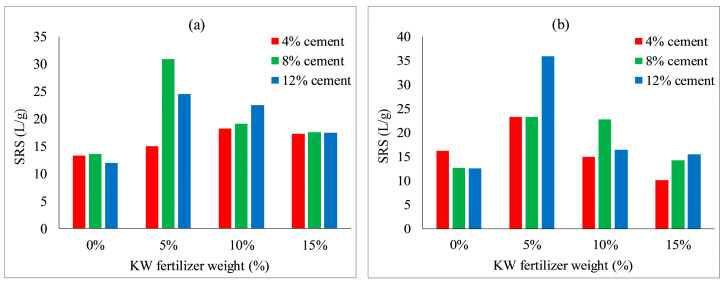
Scouring resistance of VC relative to KW fertilizer content: (**a**) *Ryegrass*; (**b**) *Festuca elata*.

Figure 17 shows that KW fertilizer content significantly affected the scouring resistance of both species. The scouring resistance of both species increased considerably when KW fertilizer content increased to 5%, achieving the highest scouring resistances of 30.9 L/g and 35.9 L/g with 8% concrete content for *Ryegrass* and 12% concrete content for *Festuca elata*, respectively. The scouring resistance decreased when KW fertilizer content increased from 5 to 15%, which may indicate that the optimum ratio of KW fertilizer content with respect to scouring resistance of VC was 5%. Meanwhile, the scouring resistances of *Ryegrass* and *Festuca elata* peaked with concrete content of 8% and 12%, respectively.

**Figure 18:**
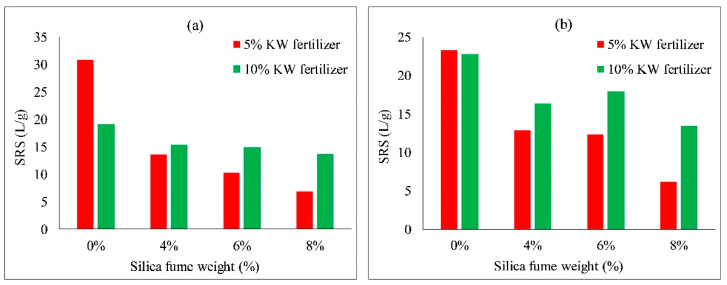
Scouring resistance of VC relative to silica fume content: (**a**) *Ryegrass*; (**b**) *Festuca elata*.

**Figure 19:**
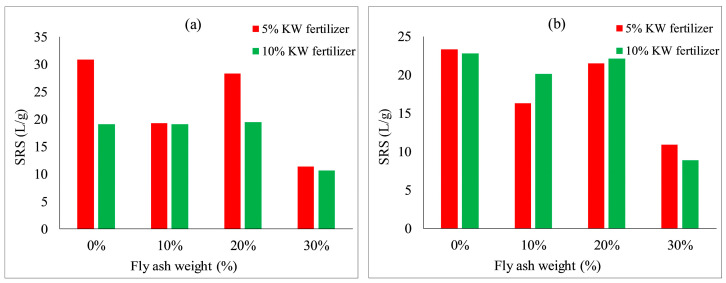
Scouring resistance of VC relative to fly ash content: (**a**) *Ryegrass*; (**b**) *Festuca elata*.

Figure 18 and Figure 19 show the effect of silica fume and fly ash contents on the scouring resistance of VC. With an increase in silica fume content from 0 to 8%, the scouring resistance of each group decreased. With an increase in fly ash content from 0 to 30%, the scouring resistance of each group fluctuated. The scouring resistances were the highest when no silica fume content nor fly ash content was added to the specimens. This may indicate the scouring resistances relative to plant heights, which mainly rely on the properties of the soil added with concrete and KW fertilizer, so the additions of silica fume and fly ash had almost no positive effect on plant height.

**Figure 20:**
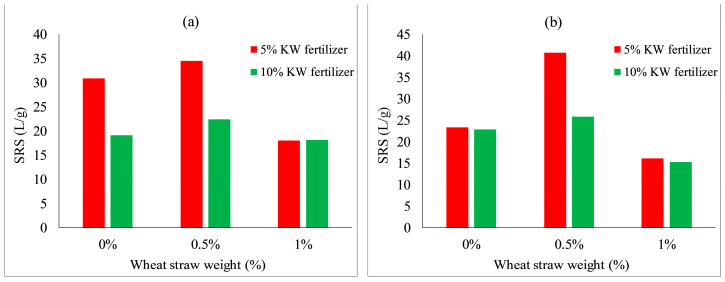
Scouring resistance of VC relative to wheat straw content: (**a**) *Ryegrass*; (**b**) *Festuca elata*.

**Figure 21:**
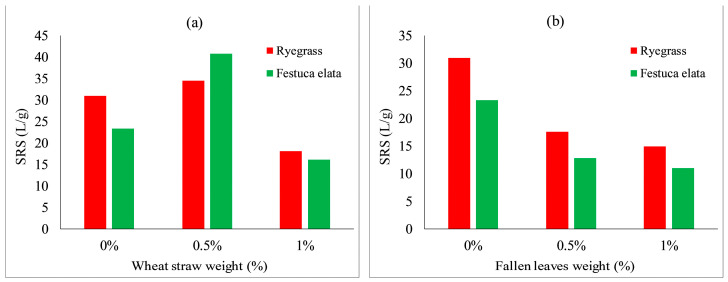
Scouring resistance of VC relative to: (**a**) wheat straw combined with 5% KW fertilizer; and (**b**) fallen leaves contents combined with 5% KW fertilizer.

Figure 20 shows the effect of wheat straw content on the scouring resistance of VC. The scouring resistance of both species increased slightly when wheat straw content increased to 0.5% and achieved the highest scouring resistances. The scouring resistance decreased slightly when wheat straw content increased from 0.5 to 1%. This may indicate that the optimum ratio of wheat straw content on the scouring resistance of VC for both species was 0.5% (as shown in Figure 21a). As depicted in Figure 21b, with an increase in fallen leaves content from 0 to 1%, the scouring resistance of both species decreased, with an inverse relation between fallen leaves content and scouring resistance.

In general, the effect of KW fertilizer content on the scouring resistance of both species was related to the cement content, which means that the SRS is influenced by the combined action of KW fertilizer and cement contents (as shown in Figure 17). Under the same KW fertilizer and cement contents, the addition of silica fume or fallen leaves decreased the SRS of both species, whereas neither fly ash nor wheat straw addition had an obvious inhibitory promoting effect on SRS. The highest SRS for both species is shown in Figure 21a, where the mix proportion of MSW-modified VC is comprised of 5% KW fertilizer, 8% cement and 0.5% wheat straw. Therefore, the combination of a moderate amount of KW fertilizer, cement and wheat straw effectively improved the SRS of the soil.

## 4. Conclusions

With the continuous development of the social economy, the establishment of sustainable materials and practices is important for future environments. Therefore, in this study, we investigated the effects of MSW (i.e., kitchen waste, wheat straw, fly ash, silica fume and fallen leaves) on vegetation characteristics of modified VC by comparative experiments. To modify plant adaptability in the environment of VC, MSW was added as an amendment to fabricate VC with different mix proportions. The effects of MSW on seed germination, grass height and the scouring resistance of different grass species were evaluated. Our results can pave the path for further research on waste management of MSW and contribute to beneficial knowledge for future research on the feasibility of the use these species with VC technology for slope ecological restoration. Our main conclusions are as follows:

(1) The designed mixtures were suitable for growing the two tested species. Most mixes had high germination rates in the first 15 days and low germination rates in the last 15 days.
(2) The germination rates and plant heights of both species decreased with an increase in concrete content; the scouring resistances of *Ryegrass* and *Festuca elata* peaked with concrete contents of 8% and 12%, respectively.
(3) Ryegrass preferred high KW fertilizer content for a remarkable plant height, whereas the optimum ratio of KW fertilizer content with respect to the scouring resistance of both species was 5%.
(4) Moderate silica fume content (e.g., <4%) had a positive effect on the plant heights of both species, whereas silica fume content had an inverse effect on the scouring resistance of both species.
(5) The addition of fly ash and wheat straw content had an inhibitory effect on plant height, but the scouring resistance of both species increased slightly when wheat straw content was increased to 0.5% and achieved the highest scouring resistances.
(6) A small amount of fallen leaves added to VC can promote the growth of *Ryegrass* somewhat and slightly for *Festuca elata*; however, fallen leaves content had an inverse effect on the scouring resistance of both species.
(7) Considering the scouring resistances, the optimal mix proportion of MSW-modified VC were recommended as No. 25 with 5% KW fertilizer, 8% cement and 0.5% wheat straw in this study.

## Figures and Tables

**Table 1:** Physical performance indices of the soil.

Relative Density	Plastic Limit (%)	Liquid Limit (%)	MDD (g/cm³)	OMC (%)
2.5	17.6	32.3	1.73	15.6

**Table 2:** Composition ratios of the MSW for VC.

Specimen No.	Material (% Weight)					
	Kitchen Waste	Cement	Silica Fume	Fly Ash	Wheat Straw	Fallen Leaves
1	0.0	4.0				
2	0.0	8.0				
3	0.0	12.0				
4	5.0	4.0				
5	5.0	8.0				
6	5.0	12.0				
7	10.0	4.0				
8	10.0	8.0				
9	10.0	12.0				
10	15.0	4.0				
11	15.0	8.0				
12	15.0	12.0				
13	5.0	8.0	4.0			
14	10.0	8.0	4.0			
15	5.0	8.0	6.0			
16	10.0	8.0	6.0			
17	5.0	8.0	8.0			
18	10.0	8.0	8.0			
19	5.0	8.0		10.0		
20	10.0	8.0		10.0		
21	5.0	8.0		20.0		
22	10.0	8.0		20.0		
23	5.0	8.0		30.0		
24	10.0	8.0		30.0		
25	5.0	8.0			0.5	
26	10.0	8.0			0.5	
27	5.0	8.0			1.0	
28	10.0	8.0			1.0	
29	5.0	8.0				0.5
30	10.0	8.0				1.0

**Table 3:** Relationship between seed germination and planting time.

No.	Number of *Ryegrass* Seed Germinations (N_1_)					Number of *Festuca elata* Seed Germinations (N_2_)				
	5 Days	10 Days	15 Days	20 Days	30 Days	5 Days	10 Days	15 Days	20 Days	30 Days
1	78	111	132	136	137	54	85	103	117	125
2	70	95	121	134	136	42	70	90	102	122
3	52	83	109	130	131	28	56	75	88	112
4	89	124	142	146	147	64	90	106	117	128
5	69	99	122	138	143	56	82	96	108	125
6	65	89	116	129	138	49	73	92	106	120
7	76	116	131	142	144	51	79	98	111	126
8	60	91	115	131	140	43	76	99	109	127
9	65	92	114	126	137	22	64	89	102	123
10	70	117	125	128	134	42	67	87	95	112
11	66	105	119	124	131	36	60	85	94	111
12	65	95	111	123	130	23	47	69	90	107
13	67	97	120	134	141	49	69	93	102	124
14	55	90	115	130	138	42	70	93	102	122
15	63	89	112	132	136	41	62	87	100	123
16	58	88	115	134	136	42	66	84	103	118
17	55	87	118	130	133	35	59	83	95	116
18	48	86	108	123	134	34	62	81	95	117
19	61	93	118	136	140	53	71	89	103	119
20	44	74	103	126	137	40	66	86	105	120
21	43	80	107	128	137	48	67	83	97	115
22	39	69	95	118	137	35	63	82	98	117
23	28	67	102	120	130	46	63	78	91	113
24	40	65	92	113	133	37	62	81	94	114
25	59	103	128	140	144	45	75	96	106	128
26	73	99	127	134	141	59	77	93	112	130
27	59	102	130	135	138	53	81	100	120	132
28	65	101	128	140	142	54	85	100	110	130
29	53	92	116	129	142	51	81	99	115	131
30	54	99	117	127	138	60	93	109	123	132

**Table 4:** Average grass heights of *Ryegrass*.

No.	Average Grass Heights of *Ryegrass* at Age (cm)							
	5 Days	10 Days	15 Days	20 Days	30 Days	40 Days	50 Days	60 Days
1	1.2	5.6	9.0	11.0	12.5	12.8	13.2	13.6
2	1.1	5.2	8.5	10.4	12.0	12.6	12.9	13.4
3	0.2	5.0	8.1	10.0	11.4	12.2	12.9	13.1
4	1.1	5.9	10.5	12.5	13.4	13.9	14.0	14.1
5	1.0	4.9	10.1	12.1	12.9	13.6	13.8	13.9
6	1.2	4.5	10.6	12.4	12.9	13.3	13.4	13.5
7	1.5	5.1	9.9	12.5	13.6	14.0	14.4	14.8
8	1.5	5.1	9.9	12.5	13.6	14.0	14.4	14.8
9	1.0	4.9	10.1	12.1	12.9	13.6	13.8	13.9
10	0.7	5.2	11.1	13.1	14.3	14.6	15.2	15.2
11	1.1	5.8	11.5	12.5	13.8	14.4	15.0	15.0
12	0.3	4.9	9.1	11.9	13.4	13.6	14.0	14.1
13	1.0	6.3	10.6	12.9	13.5	13.7	14.1	14.1
14	0.3	6.1	10.1	12.4	13.4	14.0	14.5	14.7
15	0.6	6.0	9.6	12.4	13.7	14.1	14.3	14.6
16	1.0	5.5	9.6	10.9	12.5	13.6	14.1	14.5
17	1.0	5.9	9.9	11.6	12.4	12.7	12.9	13.3
18	1.3	7.0	11.1	12.3	12.8	13.6	14.0	14.2
19	0.7	6.2	9.6	11.0	11.9	12.1	12.5	12.5
20	0.2	5.0	10.0	11.7	12.0	12.4	13.0	13.6
21	0.4	6.4	10.0	11.5	12.0	12.2	12.5	12.7
22	0.5	6.8	9.9	11.5	12.1	12.4	12.8	13.0
23	0.0	4.8	10.5	11.7	12.5	13.0	13.6	13.5
24	0.4	5.2	9.0	11.3	12.3	12.6	13.5	13.8
25	0.2	6.9	9.4	11.2	12.1	12.8	13.2	13.3
26	0.3	5.2	9.2	11.4	12.5	12.9	13.3	13.7
27	0.3	5.7	9.3	10.5	11.7	11.9	12.4	13.1
28	0.3	4.6	7.9	11.0	11.7	12.5	13.1	13.2
29	0.6	7.3	9.6	12.1	12.9	13.9	14.3	14.5
30	0.4	5.9	9.1	11.4	13.6	14.3	14.6	15.0

**Table 5:** Average grass heights of *Festuca elata*.

No.	Average Grass Heights of *Festuca elata* at Age (cm)							
	5 Days	10 Days	15 Days	20 Days	30 Days	40 Days	50 Days	60 Days
1	0.4	4.6	7.8	9.6	10.8	11.6	12.1	12.3
2	0.3	4.2	7.0	9.2	10.5	11.1	11.9	12.1
3	0.4	3.3	5.5	6.9	8.4	9.6	10.5	11.1
4	0.6	4.1	8.5	11.1	12.6	13.3	13.7	14.0
5	0.4	4.7	8.0	10.4	11.5	12.5	12.9	13.0
6	0.7	3.8	7.5	9.0	9.9	10.4	11.0	11.6
7	0.5	3.6	6.9	9.9	11.9	13.2	13.9	14.1
8	0.2	2.7	6.4	8.1	9.9	11.3	12.3	13.1
9	0.2	4.0	6.1	7.4	9.0	9.9	11.2	12.0
10	0.4	3.6	8.1	10.6	12.5	13.7	14.3	14.7
11	0.5	4.2	7.6	9.9	11.9	12.5	13.0	13.1
12	0.2	3.0	6.6	8.7	9.7	10.9	11.4	12.2
13	0.2	4.1	6.4	9.1	11.3	12.7	13.4	14.3
14	0.4	4.8	7.9	10.0	11.8	13.9	15.2	16.4
15	1.1	3.7	8.0	9.6	11.8	12.9	13.6	14.5
16	0.4	3.4	5.9	8.5	10.7	11.8	13.0	15.0
17	0.2	3.8	6.6	9.1	10.0	10.6	11.3	11.7
18	0.1	3.3	6.9	8.5	9.9	11.0	11.8	12.8
19	0.6	4.1	5.9	8.4	9.1	9.5	10.5	11.2
20	0.3	3.8	7.1	8.0	9.6	11.0	11.7	12.3
21	0.3	3.3	6.9	8.1	9.1	9.8	10.4	11.1
22	0.2	4.3	7.1	7.5	9.6	10.7	11.7	12.5
23	0.1	3.5	5.9	8.0	10.0	10.9	11.7	12.1
24	0.2	3.8	6.4	9.0	10.6	11.4	12.4	12.9
25	0.3	3.8	5.4	7.6	9.6	10.6	11.3	11.7
26	0.4	5.0	6.9	8.6	9.9	10.7	11.8	12.5
27	0.3	5.2	6.6	7.5	8.9	10.3	11.0	11.1
28	0.2	4.1	6.0	7.4	9.5	10.7	11.6	12.1
29	0.4	4.4	7.0	8.5	10.9	12.1	12.9	13.4
30	0.1	3.3	6.0	7.6	9.8	11.0	12.1	13.2

**Table 6:** SRS results after 60 days.

No.	*Ryegrass* (L/g)	*Festuca elata* (L/g)
1	13.3	16.2
2	13.6	12.6
3	12.0	12.5
4	15.0	23.3
5	30.9	23.3
6	24.5	35.9
7	18.2	15.0
8	19.1	22.8
9	22.5	16.4
10	17.3	10.1
11	17.6	14.2
12	17.5	15.5
13	13.6	12.9
14	15.4	16.3
15	10.3	12.3
16	14.9	17.9
17	6.8	6.2
18	13.7	13.4
19	19.3	16.3
20	19.1	20.1
21	28.3	21.5
22	19.5	22.1
23	11.4	10.9
24	10.6	8.9
25	34.5	40.8
26	22.4	25.8
27	18.0	16.1
28	18.1	15.2
29	17.6	12.8
30	14.9	11.0

## Data Availability

Some or all data, models or code that support the findings of this study are available from the corresponding author upon reasonable request.
